# Nucleation of Porous Crystals from Ion-Paired Prenucleation Clusters

**DOI:** 10.1021/acs.chemmater.2c00418

**Published:** 2022-06-16

**Authors:** Nick Pellens, Nikolaus Doppelhammer, Sambhu Radhakrishnan, Karel Asselman, C. Vinod Chandran, Dries Vandenabeele, Bernhard Jakoby, Johan A. Martens, Francis Taulelle, Erwin K. Reichel, Eric Breynaert, Christine E. A. Kirschhock

**Affiliations:** † Center for Surface Chemistry and Catalysis—Characterisation and Application Team (COK-KAT), KU Leuven, Celestijnenlaan 200F, 3001 Leuven, Belgium; ‡ Institute for Microelectronics and Microsystems JKU Linz, 4040 Linz, Austria; § NMR-Xray Platform for Convergence Research (NMRCoRe), KU Leuven, 3001 Leuven, Belgium

## Abstract

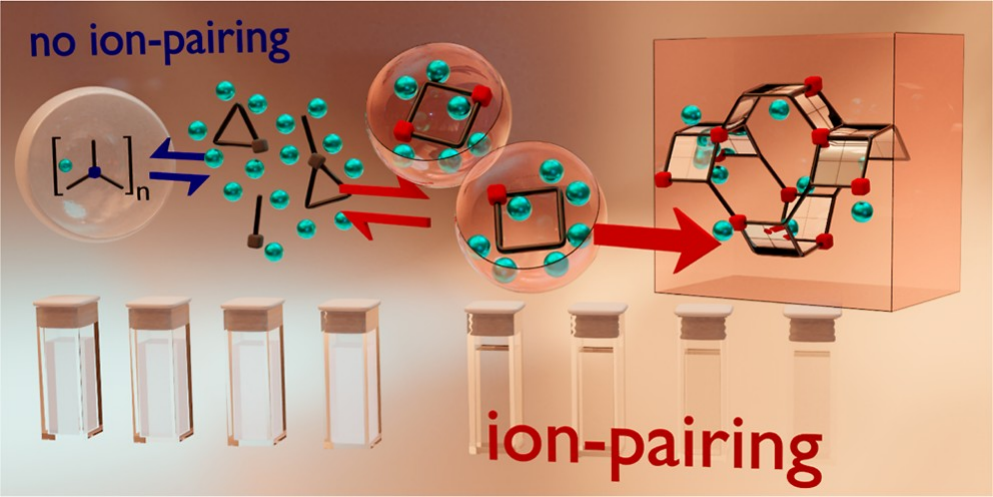

Current nucleation models propose manifold options for the formation of crystalline materials. Exploring and distinguishing between different crystallization pathways on the molecular level however remain a challenge, especially for complex porous materials. These usually consist of large unit cells with an ordered framework and pore components and often nucleate in complex, multiphasic synthesis media, restricting in-depth characterization. This work shows how aluminosilicate speciation during crystallization can be documented in detail in monophasic hydrated silicate ionic liquids (HSILs). The observations reveal that zeolites can form via supramolecular organization of ion-paired prenucleation clusters, consisting of aluminosilicate anions, ion-paired to alkali cations, and imply that zeolite crystallization from HSILs can be described within the spectrum of modern nucleation theory.

## 1 Introduction

Many solidification processes from a liquid cannot be adequately described by classical nucleation theory (CNT). The formation of crystalline nanoporous materials with structurally ordered arrays of nanoscopic pores is a prime example, where CNT does not capture nucleation. In CNT, stochastic, thermally driven density fluctuations in supersaturated homogeneous liquids result in the assembly of viable nuclei from the growth medium. Within the classical model, nucleation rates can be derived for a given degree of supersaturation, assuming invariable interfacial and bulk free energies.^1^ However, for processes involving a phase separation via metastable intermediates, nonclassical approaches are required.^2^ Metastable states, such as those arising from liquid–liquid demixing or supramolecular assemblies, not only play a role in the formation of porous crystals or biomolecular condensates^3−10^ but also can initiate crystallization of dense inorganic salts, such as calcium carbonates and calcium phosphates, or oxides such as magnetite.^11−18^ Disputing the assumptions of CNT, nucleation of these inorganic phases has been shown to proceed via condensation of prenucleation clusters.^19^ These can directly condense into crystals or pass through an amorphous prephase, depending on the surface energy of the respective solid, relative to the energy of the fluid species.^17,18,20,21^ Extending upon CNT, modern theory proposes selection between classical and nonclassical pathways.^18^ However, the specific entities involved, and their molecular-level interactions determining their condensation pathway, often remain subject to speculation. Recent advances start to reveal the role of molecular interactions such as ion-pairing in kinetic nucleation barriers, offering opportunities to also include kinetic aspects in the description of nonclassical pathways.^17,19,22^ This depends on molecular-level characterization of crystal nucleation, a significant experimental challenge, even in model systems such as the aforementioned calcium carbonates and calcium phosphates.

This work shows zeolites, a class of microporous materials with high societal relevance,^23^ to offer unsuspected opportunities to advance molecular verification of the fundamentals of non-CNT. In the early years of zeolite synthesis, Barrer, Breck, and co-workers mimicked the natural conditions in which zeolites form.^24−27^ Under natural conditions, zeolite formation spans geological timescales, but increasing alkalinity, temperature, and concentration of framework elements allowed development of heterogeneous synthesis gels yielding zeolites within hours up to days under hydrothermal conditions. Zeolite synthesis thus typically occurs in multiphasic media, obscuring experimental observation of early nucleation steps. To gain more insights into zeolite formation, alternatives to (alumino)silicate gel synthesis were developed. Among them, the so-called “clear solution” zeolite synthesis strategy uses dilute aqueous solutions of tetraalkylammonium hydroxide to nucleate zeolites under conditions more compatible with molecular characterization techniques. This approach revealed a wealth of information about the crystallization of high-silicate zeolites such as silicalite-1 and zeolite beta.^28−33^ However, while the name “clear solution” suggests these synthesis liquids to be true solutions, they are optically clear suspensions of nanosized amorphous aggregates. Research has shown that zeolite formation proceeds via these aggregates^2,7,33^ in a nonclassical manner. As a result, even in zeolite crystallization from clear solutions, zeolite crystallization from true monophasic liquids, free of any amorphous aggregates, has not yet been captured by characterization, even though the literature suggests that zeolite crystallization is driven by the liquid medium.^10^

For this reason, a new synthesis medium was developed. The reduction of the water content and the increase in the charge density in inorganic alkalisilicate systems shifts multiphasic zeolite crystallization media into the realm of clear monophasic inorganic hydrated silicate ionic liquids (HSILs), best described as room temperature melts of hydrated silicate.^34^ These liquids are accessible for characterization, and upon aluminate addition, they readily yield zeolites in a matter of hours at moderate temperatures, in the absence of any gel phase.

HSILs form upon hydrolysis of tetraethyl orthosilicate (TEOS) in stoichiometric, strongly alkaline aqueous solutions (>2.5 M MOH, M = alkali metal). Under such conditions, spontaneous coacervation separates the mixture into an ethanolic water phase and a dense, purely inorganic HSIL, free of ethanol and severely limited in water content. Alternatively, they also can be obtained by digestion of silicates in the presence of excess alkali metal hydroxides. Upon controlled addition of aluminate to such an inorganic HSIL, a transparent liquid containing hypohydrated, ion-stabilized aluminosilicate oligomers is obtained. These aluminate-doped HSILs are homogeneous and monophasic liquids containing a limited number of chemical species. Accessible by a variety of diagnostics, HSILs are ideal to acquire molecular insights into the initial steps of zeolite formation. By this way, this system can serve as a reference to study nucleation and growth of microporous frameworks in general. This work reveals that microporous crystals can be formed from prenucleation clusters consisting of ion-paired complexes, similar to those observed during mineralization of dense calcium phosphate.^15^

## 2 Experimental Section

### 2.1 Sample Preparation

Synthesis liquids were prepared based on previously reported zeolite syntheses from HSILs.^34^ Compared to traditional gel synthesis, HSIL synthesis is characterized by rigorously reduced water contents and high concentrations of alkali metal hydroxide to ensure deprotonation of (alumino)silicate species. The resulting high charge density strictly prevents gel formation.^35,36^ Preparation of HSIL synthesis media starts with the hydrolysis of TEOS in stoichiometric, highly alkaline aqueous solutions (1 TEOS (98%, ACROS Organics): 1 NaOH (>97%, ACROS Organics): 25 H_2_O (Milli-Q)). After 24 h of mechanical rotation, spontaneous coacervation separates the mixture into an ethanolic water phase and the dense, inorganic HSIL. The latter is a hypohydrated homogenous liquid, void of bulk water, containing partly hydrated alkali cations, hydroxide ions, and small, deprotonated silicate oligomers with a molar composition of 1 Si(OH)_4_: 1 NaOH: 2.1 H_2_O. Long-term stability experiments indicate that this phase is kinetically stable over periods of at least 1 year at room temperature. This dense, fully transparent, and particle-free phase is referred to as the HSIL or Na-HSIL. The zeolite synthesis liquids were prepared with a molar recipe of 0.5 Si(OH)_4_: 0.028 Al(OH)_3_: 1 NaOH: *n* H_2_O. First, NaOH pellets (>97%, ACROS Organics) were dissolved in Milli-Q H_2_O, and the solution was cooled to room temperature. Thereafter, Al(OH)_3_ (reagent grade, Sigma-Aldrich) was added and stirred until hydrolysis was complete, yielding a transparent solution of monomeric sodium aluminate. While stirring, this liquid was combined with the prepared Na-HSIL to obtain an inorganic, transparent, particle-free stock liquid with a molar composition of 0.5 Si(OH)_4_: 0.028 Al(OH)_3_: 1 NaOH: 2.5 H_2_O. Finally, NaOH zeolite synthesis liquids of 0.5 Si(OH)_4_: 0.028 Al(OH)_3_: 1 NaOH: *n* H_2_O were prepared by diluting the stock liquid with Milli-Q H_2_O to achieve the desired concentrations of *x*_NaOH_ = 1/(1.525 + *n*_H2O_) and stirred for 24 h at room temperature before analysis and zeolite synthesis. For the latter, Nalgene Oak Ridge PPCO centrifuge tubes (Fischer Scientific) with a nominal capacity of 30 mL were filled with 25.00*g* synthesis liquid and hydrothermally treated at 331.15 K for 168 h in a rotary oven. Thereafter, the samples were centrifuged at 35.000*g* for 15 min, and the supernatant phases were recovered for analysis. The precipitate was further purified via repeated dispersion–centrifugation at 35.000*g* for 15 min until the rinsing water registered a neutral pH. Solids were dried at 333.15 K and subjected to X-ray diffraction and elemental analysis.

### 2.2 Conductivity Measurements

The electric conductivity of synthesis liquids was measured with a specifically developed setup for conductivity measurements of corrosive, ionic media such as hydrated ionic liquids. High accuracy was achieved using moving electrode electrochemical impedance spectroscopy (MEEIS). A detailed description of the setup in combination with MEEIS was reported by Doppelhammer et al.^37^ In the presented work, the cell was loaded with 10 mL of the prestirred sample. Impedance spectra were measured at 20 equally spaced electrode distances between 2 and 5.75 cm. For each electrode distance, impedance values at 58 logarithmically spaced frequencies were recorded in the frequency range of 5 MHz to 5 Hz in potentiostatic mode, using a peak-to-peak amplitude of 0.1 V. Measurements were carried out at room temperature (*T* = 298.15 K), implementing 20 min of thermal equilibration to assure isothermal conditions (Δ*T* < 0.01 °C).

### 2.3 Hydrogen Electrode pH Measurements

Hydrogen electrode pH measurements were performed on a Mettler Toledo S47 SevenMulti meter, equipped with a Gaskatel pHydruinio hydrogen electrode (*T* = 295.65 K). The pH electrode consists of two platinum hydrogen electrodes, in contact with a H_2_ saturated atmosphere, directly probing the pH-dependent potential of the H_2_ oxidation reaction, H_2_ → 2H^+^ + 2e^–^. Given the intrinsic linear dependence between [H^+^] and *E*_rel_, the internal buffer pH is assessed via one-point calibration using a pH = 7 calibration buffer (Gaskatel), resulting in the simplified relation between the calibrated potential (*E*_rel_) and the pH
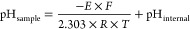


### 2.4 Dynamic Light Scattering Measurements

Dynamic light scattering measurements were performed on an LS Instruments spectrometer, equipped with a 660 nm laser at room temperature. This setup features a 320-channel correlation with a delay time of 12.5 ns to 15 h for auto- and 3D cross-correlation, recorded with a dual avalanche photodiode detector (QE = 65%, max. 250 dark counts/s). During measurement, borosilicate cylindrical measurement cells with the liquid sample were submerged in an index-matched container with decaline. The liquid colloid content was qualitatively assessed via multiple scattering, comparing measurements in the autocorrelation and 3D cross-correlation mode. To allow proper detection of colloids in the autocorrelation mode, samples were filtered through a 200 nm pore size hydrophilic PFTE membrane.

### 2.5 X-ray Diffraction Measurements

Laboratory high-resolution PXRD patterns were recorded on an STOE STADI MP diffractometer (CuKα_1_ radiation), with a focusing Ge(111) monochromator in Debye–Scherrer geometry, with a linear position sensitive detector (internal resolution 0.01°) at room temperature. Powders were ground in a mortar and transferred to glass capillaries (0.5 mm inner Ø). XRD patterns were recorded for capillaries with known packing densities of the solids. After identification of the solid and elemental composition, absorption corrections were applied, and Bragg-crystalline and amorphous fractions were quantified with WinX^POW^ software.

### 2.6 ICP-OES Measurements

The aluminate contents of synthesis and supernatant liquids and synthesized solid products were measured on an axial simultaneous ICP-OES instrument (Varian 729-ES) with a cooled cone interface and oxygen-free optics. The instrument was calibrated with an internal standard prepared from commercial silicon and aluminum standard solutions (Sigma-Aldrich) to achieve a linear signal response region between 0 and 10 ppm Al. All liquid samples were diluted with 0.42 M HNO_3_ (65%, ACROS organics) to fall within this region. Solid products were digested via the muffle-furnace method: 50 mg of the solid product was combined with 250 mg of LiBO_2_ (>99.9%, Sigma-Aldrich), dissolved in 50 mL of 0.42 M HNO_3_, transferred to a muffle furnace (Nabertherm) at 1273.15 K for 10 min, and further diluted with 0.42 M HNO_3_ to an estimated Al concentration of 0–10 ppm to ensure a linear response.

### 2.7 ^1^H, ^23^Na, ^27^Al, and ^29^Si MAS NMR Measurements

^1^H, ^27^Al, and ^23^Na measurements were performed on a Bruker Avance III 500 MHz spectrometer equipped with a 4 mm H/X/Y triple resonance magic-angle spinning (MAS) probe. The samples were filled in a disposable Kel-F insert and inserted into a 4 mm ZrO_2_ rotor. ^1^H direct excitation MAS spectra were acquired at 3 kHz, using 30° flip angle pulses at 83 kHz, a recycle delay of 5s, and averaging 32 transients. The spectra were referenced to a secondary reference, adamantine, which was further referenced to the neat tetramethyl silane (TMS). Direct excitation ^27^Al MAS NMR spectra were acquired at 3 kHz using a 45° flip angle pulse at 57 kHz, 1s recycle delay, and averaging 1024 transients. Chemical shift referencing was performed with respect to a solution containing 0.1 M Al(NO_3_)_3_ dissolved in 0.1 M HNO_3_. Direct excitation ^23^Na MAS NMR spectra were acquired at 3 kHz using a 45° flip angle pulse at 54 kHz, a 1s recycle delay, and averaging 256 transients. The spectra were referenced to 0.1 M NaCl solution in D_2_O. ^29^Si NMR measurements were performed on a Bruker Avance III 300 MHz spectrometer equipped with a 4 mm H/X double-resonance MAS probe. The rotor with the sample in the Kel-F insert was spun at 4 kHz. Direct excitation ^29^Si MAS NMR spectra were acquired with a pi/2 pulse at 66 kHz, a recycle delay of 20 s, averaging 8000 transients, and implementing ^1^H spinal-64 decoupling at 17 kHz during acquisition. The spectra were referenced to Q8M8 as a secondary reference, which was further referenced to neat tetramethylsilane (TMS). Spectral decomposition was performed with Python, employing the lmfit curve-fitting package.^38^

## 3 Results

To elucidate the molecular interactions governing zeolite nucleation from HSILs, a sample series with the composition 0.5 Si(OH)_4_: 0.028 Al(OH)_3_: 1 NaOH: *n* H_2_O was studied. A high but identical Si/Al ratio was chosen for all synthesis mixtures, and the water content was varied from *n* = 2.5 to 300. Figure 2a shows a phase diagram indicating the studied samples next to the regimes of dilute aqueous solutions and gels in function of the molar fractions of Si(OH)_4_ and Al(OH)_3_, H_2_O and NaOH (e.g., *x*_NaOH_ = *n*_NaOH_/(*n*_NaOH_ + *n*_H_2_O_ + *n*_Si(OH)_4__ + *n*_Al(OH)_3__)). The synthesis liquids were extensively characterized by conductivity, hydrogen pH, DLS, and ^1^H, ^23^Na, ^27^Al, and ^29^Si MAS NMR measurements (Sections 3.1 and 3.2) prior to synthesis. Then, all mixtures were sealed and heated to 60 °C. A synthesis time of 7 days proved sufficient to fully complete the crystallization (Section 3.3). The solid products were recovered by centrifugation, dried, and characterized by quantitative powder X-ray diffraction. The supernatant liquids were subjected to elemental analysis using ICP-OES.

### 3.1 Phase Separations in HSIL Synthesis Liquids

**Figure 1:**
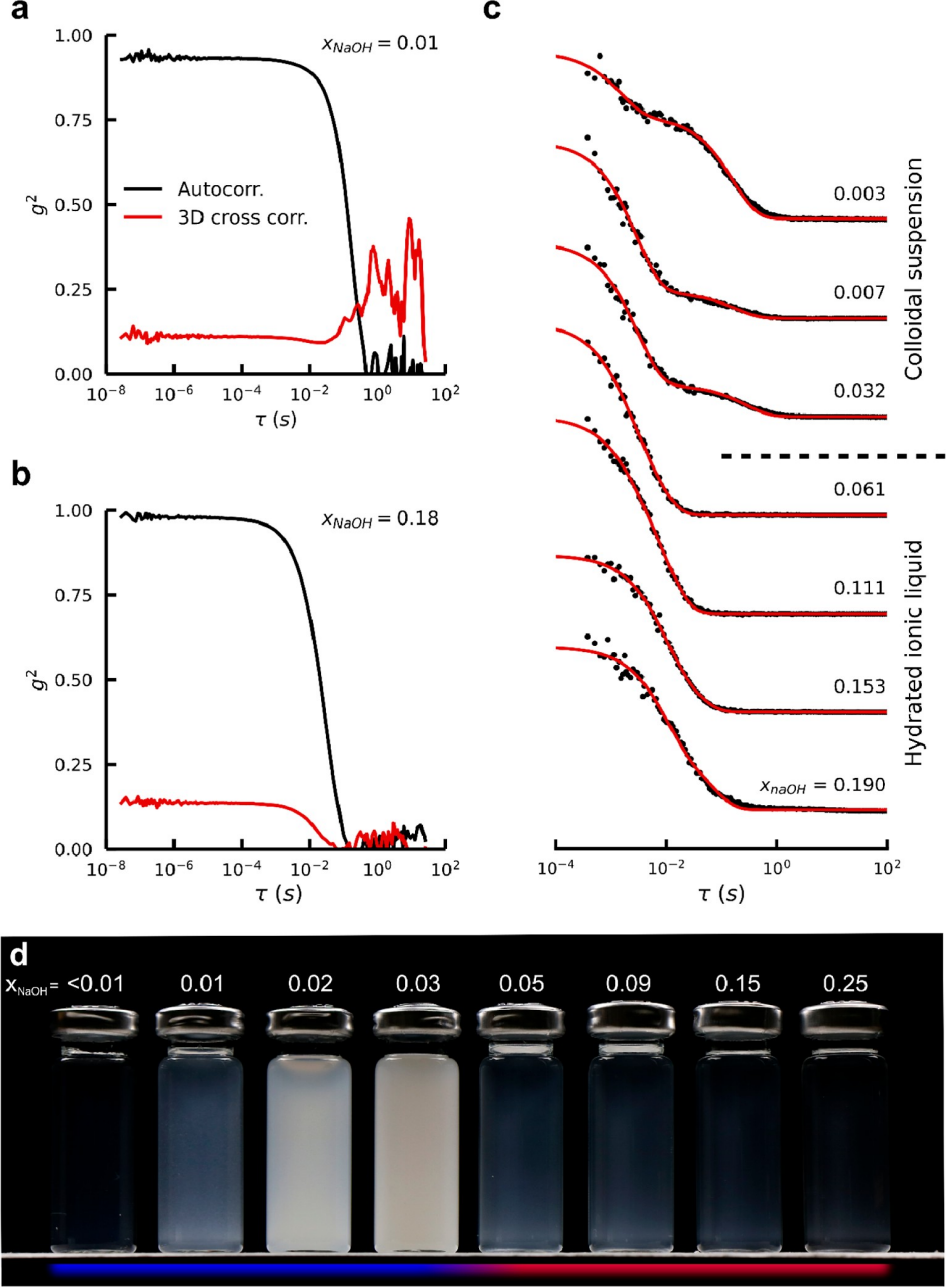
Phase separations in HSIL synthesis liquids. (a,b) Comparison of autocorrelation and 3D cross-correlation dynamic light scattering measurements. (c) Autocorrelation DLS measurements for zeolite synthesis liquids with various water contents, filtered over a 200 nm pore size hydrophilic PFTE membrane. (d) In the hydrated ionic liquid domain, synthesis liquids are clear as ion-pairing stabilizes aluminosilicate species in the liquid phase. Upon dilution, the liquid becomes increasingly turbid, which is especially pronounced when *x*_NaOH_ < 0.05, indicating the formation of a colloidal suspension, as verified by DLS measurements.

Upon increasing dilution of the HSIL synthesis liquid, a dramatic turbidity change occurs at *x*_NaOH_ < 0.05 (Figure 1d). Although even samples at higher concentrations scatter some light, comparison of autocorrelation DLS (acDLS) and 3D cross-correlation DLS (ccDLS) data (Figure 1a,b) revealed that these samples did not contain colloidal scatterers. Both correlation methods agree, suggesting local density fluctuations as a cause for the slightly turbid appearance. The turbid samples, however, clearly show the presence of colloids. In this regime (*x*_NaOH_ < 0.05), the turbid suspensions and even the clearly appearing diluted solutions show additional DLS signatures, typical for colloids (Figure 1c).

### 3.2 Characterization of HSIL Synthesis Liquids

**Figure 2:**
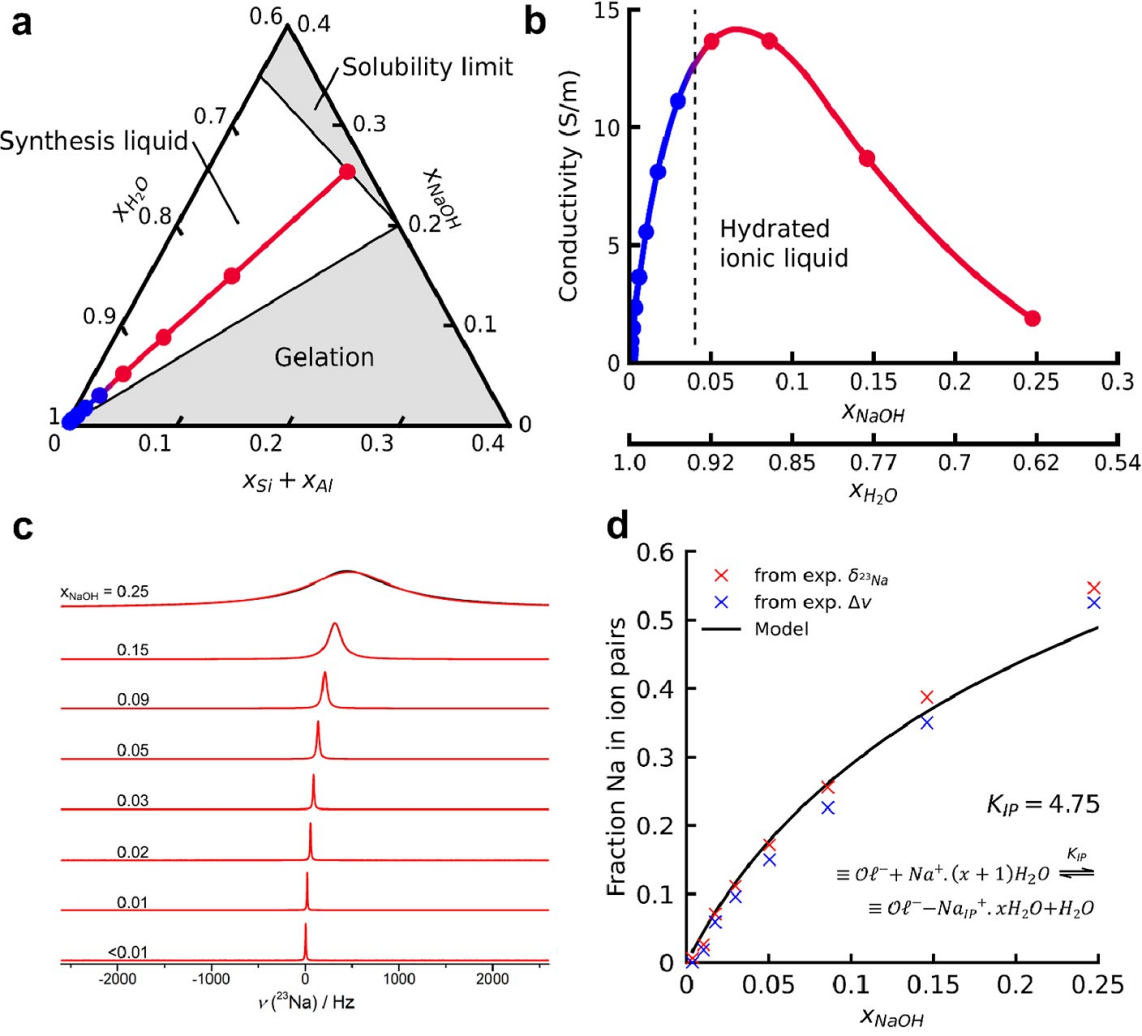
Ionic interactions in HSIL zeolite synthesis liquids. (a) Ternary diagram showing the compositional borders of liquid-phase synthesis mixtures. The transition from red to blue data points shows the transition from the hydrated ionic liquid to colloidal suspension domain. (b) Conductivity measurements of zeolite synthesis liquids of variable water contents (corresponding to the line in (a)) reveal a conductivity maximum at *x*_NaOH_ ∼ 0.06. The dashed line indicates the transition, with increasing dilution, from the hydrated ionic liquid to colloidal suspension. (c) ^23^Na exchange modeling performed based on the identified λ_IP_ = 6.18 ppm from chemical equilibrium modeling. (d) Quantification of fraction sodium cations in ion pairs by ^23^Na NMR.

Prior to zeolite crystallization, the ionic interactions in the synthesis mixtures were probed by conductivity measurements. A novel measurement principle and custom-made cell enabled high-precision conductivity measurements of the corrosive HSIL synthesis liquids.^37,39,40^ The nominal charge density of a sample can be expressed as a function of its molar fraction of NaOH (*x*_NaOH_). With reference to infinite dilution, the conductivity of NaOH solutions increases with decreasing water content because the concentration of mobile, fully hydrated ions increases. At *x*_NaOH_ ≥ 0.05, however, a further increase in the NaOH concentration leads to a conductivity decrease despite an increase in the nominal charge density. This behavior is well-known for concentrated electrolyte solutions, indicating that ion-pairing is occurring, caused by the unavailability of cation-solvation partners.^41^ In the present case, ion-pairing is provoked by water deprivation. Compared to free, solvated ions, ion pairs show significantly reduced ionic mobility and lower response to electric fields, therewith decreasing conductivity of the electrolyte (Figure S1). Note that the transition from clear liquids to turbid colloidal suspensions (Figure 1) coincides with the maximum in conductivity (Figure 2b). Therefore, the phase transition from clear liquid to colloidal suspension upon increasing the water content is best described by the transition from a homogeneous ionic liquid to an aqueous colloidal suspension.

In the studied HSIL zeolite synthesis media, the experimentally observed ion-pairing above *x*_NaOH_ ≥ 0.05 implies intimate interaction between Na^+^ and either hydroxide or (alumino)silicate anions. Comparing the conductivities of the synthesis media to pure NaOH solutions with equal nominal charge density reveals that hydroxide participation is negligible or fully absent in HSIL zeolite synthesis media. Accurate pH measurements (Figure S2), employing a combined SHE setup, confirm a low concentration of free hydroxide ions in the liquids, revealing that up to 95% of the initially present hydroxide has been used for silanol dissociation, so that, on average, each silicon center is 1.9 times deprotonated. Furthermore, the measured hydroxide content in the synthesis media remains constant upon moderate dilution (Figure S2). This infers preference of sodium for association with (alumino)silicate anions rather than with hydroxide ions, in agreement with the conductivity measurements and in line with the literature.^42^

Due to fast chemical exchange of ^23^Na, the chemical shift, as observed via quantitative ^23^Na MAS NMR measurements,^43^ is a superposition of all possible states, which allows the use of superposition theory to interpret the ^23^Na chemical environment^42^
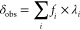
with *f*_*i*_ being the fraction of Na nuclei within the free (*f*_free_) or ion-paired state (*f*_IP_) and λ_free_ or λ_IP_ being the chemical shift value of a nucleus within that respective state. In line with the work of McCormick,^42^ a chemical equilibrium model is employed to describe the evolution of fractions of free (*f*_free_) and ion-paired (*f*_IP_) sodium nuclei in function of the synthesis liquid composition
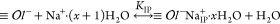


For describing the chemical environment of sodium nuclei, no differentiation between ion pairs with negative charges on silicate or aluminate ( = Si–O^–^ or Al–OH^–^) in the oligomers is made. Based on the pH measurements (Figure S2), a complete silanol deprotonation is assumed. Therefore, the initial concentrations of  anions (), sodium cations (*x*_Na^+^,init_ = *x*_NaOH_), water (*x*H_2_O,init = 1–1.528 × *x*_NaOH_), and ion pairs (*x*_IP,init_ = 0) can be deduced. In equilibrium, accounting also for explicit water, the fractions of free and ion-paired sodium cations are given by
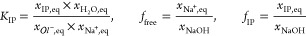


Based on the experimentally observed ^23^Na chemical shift (δ_obs_, Table S4), λ_free_ is assumed to be negligible, reducing the superposition model to
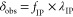


Employing the ChemPy Chemistry and SciPy^44^ Optimize modules, the superposition model is fitted to the experimental data for unknown values of *K*_IP_, determining *f*_IP_, and λ_IP_. As observed in Figure 2d, this ion-pairing model can describe the observed trend, yielding *K*_IP_ = 4.75 and λ_IP_ = 6.18 ppm. Additionally, due to quadrupolar relaxation of ^23^Na nuclei, when sodium is involved in complexes with a considerable lifetime, the line width (Δ*v*) represents the electric field gradient of these complexes^45^
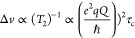


Therefore, the chemical model fitting results can be further tested via a ^23^Na exchange simulation (Figure 2c) using Bloch–McConnell equations for two-site chemical exchange.^46^ As shown in Figure 2d, the exchange model is in good agreement with the recorded spectra, yielding highly similar values for *f*_IP_.

Liquid-state ^27^Al and ^29^Si MAS NMR measurements are performed to elucidate aluminosilicate oligomer speciation. For identification, the ^29^Si analysis is restricted to single-phasic samples within the homogeneous ionic liquid domain (Figure S4). ^29^Si spectra of samples at higher dilutions, containing also colloids, were not quantified due to the low sensitivity of ^29^Si MAS NMR and the complexity associated with multiphasic liquid samples (Figure S4). These measurements reveal six categories of silicate contributions. Based on observations of silicate oligomerization in sodium silicate solutions, monomeric, three-ringed, and four-ringed silicate oligomers are identified to dominate.^9^ Prior literature on potassium aluminosilicate solutions^47,48^ shows two additional oligomeric contributions belonging to aluminosilicate dimers, branched and unbranched three rings.

**Figure 3:**
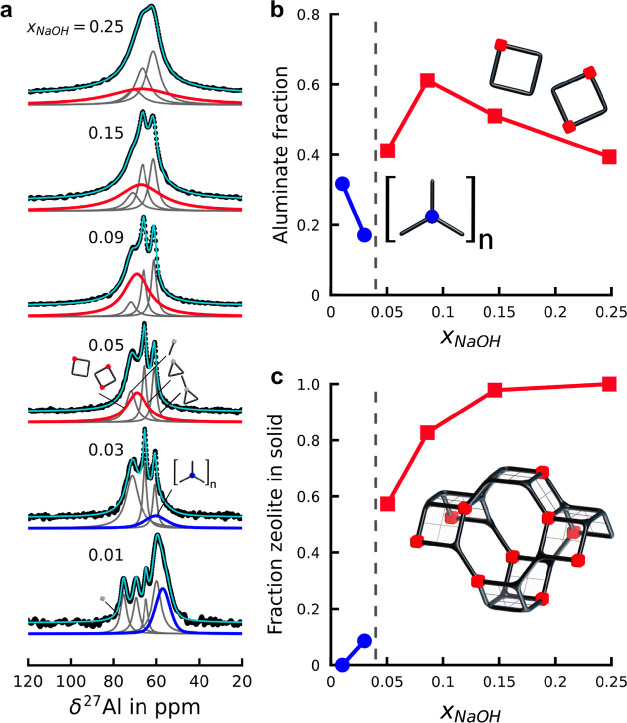
Aluminosilicate anion–sodium cation ion pairs initiate zeolite formation from homogeneous liquids. (a) Visual overview of fitted ^27^Al MAS NMR spectra. Individual fits and fitting parameter trends are shown in Figures S5 and S6. Highlighted red and blue contributions resemble the aluminate in the prenucleation complex and colloidal fractions, respectively. (b) Relative contribution of various aluminosilicate species in recorded ^27^Al MAS NMR spectra. For plotting clarity, the species present in all samples, including aluminosilicate dimers and (un)branched three rings, were not shown. (c) Quantitative analysis of the XRD measurements, based on the total Bragg reflection surface after absorption correction and background subtraction.

As shown in Figure 3a, ^27^Al spectra are robustly deconvoluted into the individual contributions. Due to troublesome signal overlap, it was not possible to deconvolute the ^27^Al spectrum of *x*_NaOH_ = 0.02, which is not included in the analysis (Figure S5). The fitting parameters are included in Table S5 and Figure S6. All contributions have a Lorentzian line shape, except for a single Q3 ^27^Al signal, exclusively observed for samples *x*_NaOH_ = 0.03 and 0.01. As discussed above, diluting the synthesis liquids triggers a phase separation at high water content (*x*_NaOH_ < 0.05). In ^27^Al MAS NMR, this can be observed via a Gaussian character in the line shape, as indicated in blue in Figure 3. In addition, an increase in average liquid to solid connectivity from ∼2 to 3 is observed (Figure S6), in line with nanoaggregate formation in silicalite “clear solution” zeolite synthesis liquids.^49,50^ Therefore, this contribution is ascribed to Q3 aluminosilicate nanoaggregates.

Apart from the nanoaggregates, the ^27^Al NMR spectra consist of the Q1 aluminosilicate dimer, Q2 unbranched aluminosilicate three rings, and Q3 branched aluminosilicate three rings with comparable line widths (Figure S5). In the ionic liquid domain (*x*_NaOH_ ≥ 0.05), in the absence of nanoaggregates, another Q2 contribution is observed next to the collection of aluminosilicate oligomers. Interestingly, this contribution is the dominant aluminosilicate oligomer and displays a significantly increased line width compared to other species (Figure S6). Based on chemical shift, this Q2 contribution could belong to aluminate centers in linear or four-ring Q2 aluminosilicate oligomers. A study on kinetics of exchange within aluminosilicate solutions suggests that cyclic species are significantly more stable compared to acyclic variants, showing slower exchange.^47^ Additionally, a series of simulation studies investigated the stability and condensation of aluminosilicate species in close interaction with sodium cations.^51,52^ Such a close interaction with sodium cations strongly favors cyclic oligomers via bond angle relaxation. Finally, both simulation^53^ and experiment^54^ postulated aluminosilicate four-ringed oligomers as crucial species for zeolite nucleation and growth. Based on these findings, we ascribe the signal at 66–69 ppm to aluminosilicate four rings, in close interaction with sodium cations.

### 3.3 Characterization of Synthesis Products

**Figure 4:**
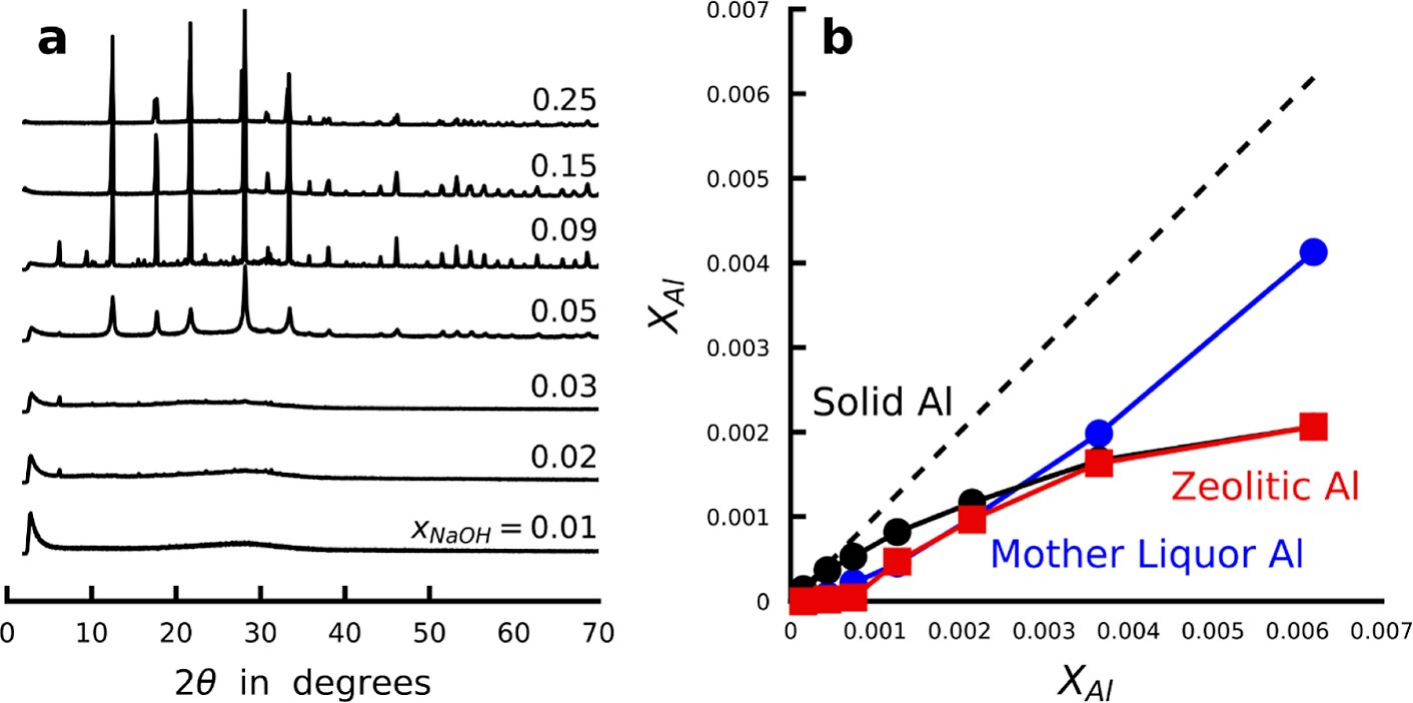
Quantitative zeolite synthesis yield analysis. (a) Quantitative X-ray diffraction measurements of synthesized solid synthesis products of synthesis liquids with water contents of *x*_NaOH_ = 1/(1.528 + *n*_H2O_), resembling nominal molar compositions of 0.5 Si(OH)_4_: 0.028 Al(OH)_3_: 1 NaOH: *n* H_2_O. (b) Combining quantitative X-ray diffraction with ICP-OES elemental analysis allows a quantitative zeolite yield analysis.

Synthesis liquids with high NaOH concentration (*x*_NaOH_ ≥ 0.05 and *x*_H2O_ ≤ 0.95; cf. Figure 2a) formed high-quality GIS-type zeolite, as identified by XRD (Figure 4a). For samples *x*_NaOH_ = 0.09 and 0.05, a trace of FAU-type zeolite was detected. The solids formed in the less-concentrated systems, where colloidal aggregates are present at room temperature, were found to be largely amorphous. Quantitative X-ray diffraction measurements were performed with capillaries with known packing densities. In combination with ICP-OES elemental analysis on the synthesized solids (Tables S1 and S2), absorption corrections were applied. Thereafter, the crystalline fractions (*f*_zeolite_) were obtained by comparing Bragg-scattered intensity to total recorded intensity over the whole measurement range (Figure 3c). By choosing a high Si/Al ratio of 18 in all synthesis mixtures, Al depletion of the liquid (*f*_Al,used_), measured via ICP-OES elemental analysis, can be compared to the elemental composition of the total product and the zeolitic fraction therein. In combination with *f*_zeolite_, this allows expression of the zeolite synthesis efficiency in terms of the total aluminate content (*x*_Al_), being the sum of the aluminate remaining in the mother liquor (*x*_Al,ML_) and present in the formed solid (*x*_Al,solid_). The latter includes the quantitative zeolite yield (*x*_Al,zeolite_) based on aluminum (Figure 4b).

## 4 Discussion

Remarkably, zeolite formation in the here-studied system is observed exclusively when the synthesis liquids are dominated by ion-pairing between aluminosilicate and alkali cations, in the absence of amorphous colloids. Such colloids in the synthesis liquids are observed, but only at high water content. This suggests that water, when available as a coordination partner for sodium, prevents the stabilization of the small, ionic aluminosilicate oligomers, which allows the formation of nanoaggregates with a high aluminum content. The here-chosen sample series covers the changing character of the synthesis liquids from colloidal aqueous suspension to ion-paired hypohydrated ionic liquids (Figure 2). All observations imply that the solubility of aluminate decreases with increasing water content, a fact which on first sight might appear counterintuitive. However, in water-deficient systems, with their negligible presence of sodium hydroxide ion pairs, virtually all (alumino)silicate oligomers, including all potential zeolite precursors, take part in ion-pairing with alkali cations. This efficiently suppresses nanoaggregation. The multidiagnostic characterization of the ion-paired synthesis liquids implies that these systems consist of a dense network of sodium coordination polyhedra, where the solvation sphere around sodium ions mainly consists of charge-compensating (alumino)silicate oligomers next to the few available water molecules and hydroxides. Such an arrangement is reminiscent of the dense polyhedral networks in concentrated sodium hydroxide liquids.^55,56^

The characterization of the solid synthesis products identifies aluminate, with its speciation and solubility in the synthesis medium, as the determining element for nucleation and growth of aluminosilicate zeolites, in line with earlier reports thereof.^23,34,48^ Liquid-state ^27^Al MAS NMR measurements (Figure 3a) indicate that high charge density and scarcity of water in the ionic liquid regime lead to aluminate incorporation into small, ion-paired aluminosilicate oligomers. Aluminosilicate dimers and (un)branched three rings are observed in all samples. However, in the here-studied concentration series, aluminosilicate four rings are exclusively observed within the homogeneous ionic liquid domain (Figure 3b). Furthermore, the observed line widths of the ^27^Al NMR signal arising from aluminosilicate four rings is particularly sensitive to the extent of ion-pairing with sodium cations (Figures 2c, 3b, and S6), suggesting that these species are in close and persistent contact. The transition from liquid-borne four-ring aluminosilicate, ion-paired with sodium, to colloidal aluminosilicate nanoaggregates at higher water contents clearly marks the change from crystalline zeolite to amorphous products, precipitating during synthesis (Figure 3c).

These observations can be brought into context with recent advances in general nucleation theory.^2,12,18,19^ Baumgartner et al. demonstrated the existence of multiple pathways to form a crystalline phase from its primary components.^18^ Depending on the physical state and the nature of their growth units, the thermodynamic energy barriers for the formation of ordered solids are vastly different. For example, the initial formation of an amorphous bulk phase can interfere with the direct formation of a crystalline product. In other words, condensation of primary components into a nonordered phase competes with direct crystallization of these components into an ordered crystal. From a thermodynamic perspective, it is concluded that pathway selection is governed by the surface-to-bulk energy ratio of the involved species.^18^ Solidification via an amorphous intermediate (nonordered condensation) is favorable when this ratio is low, while high surface energies prevent this intermediate step, enforcing direct crystallization via ordered condensation.^18^

In the here-studied systems, zeolite formation only proceeds in the presence of liquid-borne aluminosilicate four-ring-alkali ion pairs (Figure 3). In the more diluted syntheses, amorphous aluminosilicates form, but subsequent zeolite formation by reorganization and densification of this phase is severely limited during the moderate hydrothermal treatment. In the higher concentrated, ionic liquid systems, our observation of zeolite formation, exclusively in the presence of specific oligomer–cation pairs implies a significantly lower energy barrier for direct zeolite formation compared to the formation of an intermediate amorphous product. Although amorphous colloidal nanoaggregates can be formed in more diluted systems, ion-pairing, in the case of high charge density, increases the surface energy of any solid phase, thus forcing the system to remain in the liquid state and strictly preventing gel formation.^35,36^ From there, only solids with higher thermodynamic stability of the bulk compared to amorphous solids can emerge. Consequently, the formation of crystalline, ordered zeolite products agrees with the predictions of Baumgartner, favoring the transition from nonordered to ordered condensation in the case of increasing surface energy.^18^

On a molecular level, the aluminosilicate sodium ion-association complexes follow the major characteristics of prenucleation clusters, as defined by Gebauer et al.^19^ To enable solid formation, prenucleation clusters should be dynamic, liquid-borne molecular fragments resembling the structure and energy of the bulk crystal.^19^ The here-discovered ion pairs contain aluminosilicate four rings in interaction with cations and their hydration water, similar to that found later in the final GIS-type zeolite, even though their exact configuration may change during initial condensation, nucleation, or attachment to a growing crystal, just as required by theory.^19^

**Figure 5:**
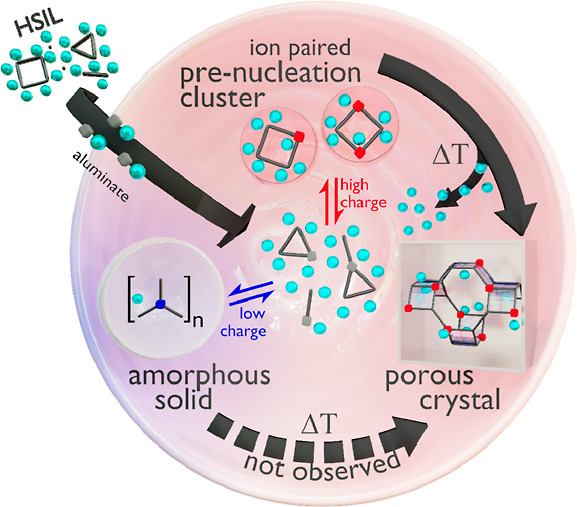
Schematic overview of porous crystal formation from prenucleation ion pairs. Inspired by Baumgartner et al., reporting on the nucleation of magnetite.^18^

Fully consistent with Gebauer et al.^19^ and the present work, Anderson et al.^12^ recently demonstrated that a 3D crystal partition model can describe crystal growth for a wealth of porous materials, including zeolites and MOFs. While the growth of porous materials is highly complex, condensation of molecular fragments onto a growing porous surface must follow simple rules, favoring condensations resulting in cage closure that lower the crystal surface energy.^12^ The aluminosilicate four-ring-alkali ion pair which was identified as a prenucleation cluster for GIS zeolite formation (Figure 4) qualifies as such a conceptual unit as the GIS topology can be thought of as being constructed entirely from the condensation of single aluminosilicate 4 rings (4rs). However, despite the qualification of the ion-paired aluminosilicate 4r as prenucleation clusters for the formation GIS-type zeolite, there are differences in its role, compared to the prenucleation concept described in the literature. Although there, prenucleation clusters imply a loose assembly of growth units prior to nucleation, in the here-studied case, it needs to be considered that the ion-paired 4r species observed by NMR are in constant chemical exchange with all other aluminosilicate species in the liquid. The liquid state can best be perceived as a polyhedral network of cations ion-paired to constantly interconversing oligomers. Nonetheless, the solubility of the different aluminosilicate species should differ, and indeed, an additional experiment shows that GIS-type zeolite synthesis only proceeds in the case of supersaturation of these ion-paired 4rs (Figure S7). This observation establishes a direct link between the occurrence of prenucleation clusters in the liquid state (Figure 3b) and their likelihood to leave the liquid phase via nucleation and crystal growth^19^ (Figure 3c). A recent kinetic study of this system also reveals that the growth of GIS-type zeolite from ionic liquids, at temperatures slightly higher than 60 °C, seems to resemble the classic nucleation theory pathway because no induction period prior to crystallization is observed.^57^ However, a clear classification as CNT growth is problematic. Assuming the cation-4r prenucleation clusters as the growth unit, crystal growth must occur incongruently because excess cations and hydroxide generated by condensation need to be released during growth. Furthermore, the ease by which the nucleation and growth can be steered toward amorphous products by destabilization of the prenucleation clusters indicates that the system is best described considering kinetic and thermodynamic aspects. In other words, our molecular-scale investigation of homogeneous zeolite synthesis from HSILs demonstrates that modern nucleation theory can successfully also describe the formation of a porous crystal, as schematically shown in Figure 5. Even though the here-discussed synthesis media are highly different from the typical gel syntheses for small- and medium-pore zeolites, they offer a promising alternative for their synthesis as demonstrated by the efficient crystallization of at least 14 different small- and medium-pore aluminosilicate zeolites from HSIL media.^58^ Although not directly transposable, the insights gained from zeolite crystallization in these highly ionic media contribute important clues for zeolite formation from gel systems. Despite the cumbersome molecular-level characterization, also in gel systems, growth has been suggested to occur via the liquid medium.^10^ The present study reveals that the supersaturation of specific aluminosilicate oligomers ion-paired with cations is essential for successful zeolite formation in HSILs. Interestingly, a recent study by our group reports on systems containing nanoaggregates at room temperature which produce amorphous products at 60 °C but reliably yield the GIS-type product at 90 °C.^58^ In this study, it was shown that nucleation and growth occur in the liquid phase and that the amorphous fractions dissolve over time in favor of the crystalline product. Apparently, the increased temperature enables to overcome the nucleation barrier of the crystalline product, fully in line with the concept of modern nucleation theory. In addition, the here-reported concept of zeolite crystallization via ion-paired prenucleation clusters allows us to derive molecular kinetic crystallization models, describing zeolite growth, crystal size, and morphology.^57^ Further work is, however, needed to relate these observations to synthesis compositions prevailing in typical gel systems.

## 5 Conclusions

Current nucleation theory largely relies on thermodynamic arguments. The transformation of small liquid-borne ion-paired prenucleation complexes into ordered, larger species, eventually undergoing phase separation into viable nuclei, however, must involve several processes subject to physicochemical kinetics and giving rise to kinetic nucleation barriers. Options could include the removal of excess charge and hydration water, as well as restoration of the removed cations’ coordination environment with remaining water or anionic ligands. It is therefore speculated that besides molecular-level understanding and identification of specific zeolite prenucleation clusters, also the mechanism for their rearrangement into condensed species is required to gain insights into zeolite polymorphism and enable rational zeolite design. Such an understanding gained on the specific example of zeolite growth from HSIL media may also provide insights into the transition between the growth mechanisms governing zeolite formation in solution-mediated systems and even gels, used for commercial, nonequilibrium synthesis protocols.^10,23^ In extension, this work demonstrates that the crystallization of microporous materials follows the same thermodynamic and kinetic rules as that developed for dense crystal systems. Molecular-level investigations, however, are crucial to gain a detailed understanding of a specific crystallization pathway for any given microporous material.

## Supporting Information Available

Liquid- and solid-state characterization methods and data as referenced in the manuscript, including conductivity data, NMR spectra, pH measurements, and mass recipes of synthesis mixtures (PDF)
