# Supplementary material for: Nucleation of Porous Crystals from Ion-Paired Prenucleation Clusters
Source: Chem Mater. 2022 Jun 16;34(16):7139–49. doi: 10.1021/acs.chemmater.2c00418 (PMC9404542; doi:10.1021/acs.chemmater.2c00418)
Supplement: Supplementary file 1 — cm2c00418_si_001.pdf [file cm2c00418_si_001.pdf]

# Nucleation of porous crystals from ion-paired pre-nucleation clusters

Nick Pellens[1], Nikolaus Doppelhammer[1,2], Sambhu Radhakrishnan[1,3], Karel Asselman[1], C. Vinod Chandran[1,3], Dries Vandenabeele[1], Bernhard Jakoby[2], Johan A. Martens[1,3], Francis Taulelle[1,3], Erwin K. Reichel[2], Eric Breynaert[1,3,*], Christine E.A. Kirschhock[1]

[1] Center for Surface Chemistry and Catalysis – Characterisation and Application Team (COK-KAT), KU Leuven, Celestijnenlaan 200F, 3001 Leuven, Belgium
[2] Institute for Microelectronics and Microsystems JKU Linz, 4040 Linz, Austria
[3] NMR-Xray platform for Convergence Research (NMRCoRe), KU Leuven, 3001 Leuven, Belgium

*Corresponding author: eric.breynaert@kuleuven.be

# Supplementary information

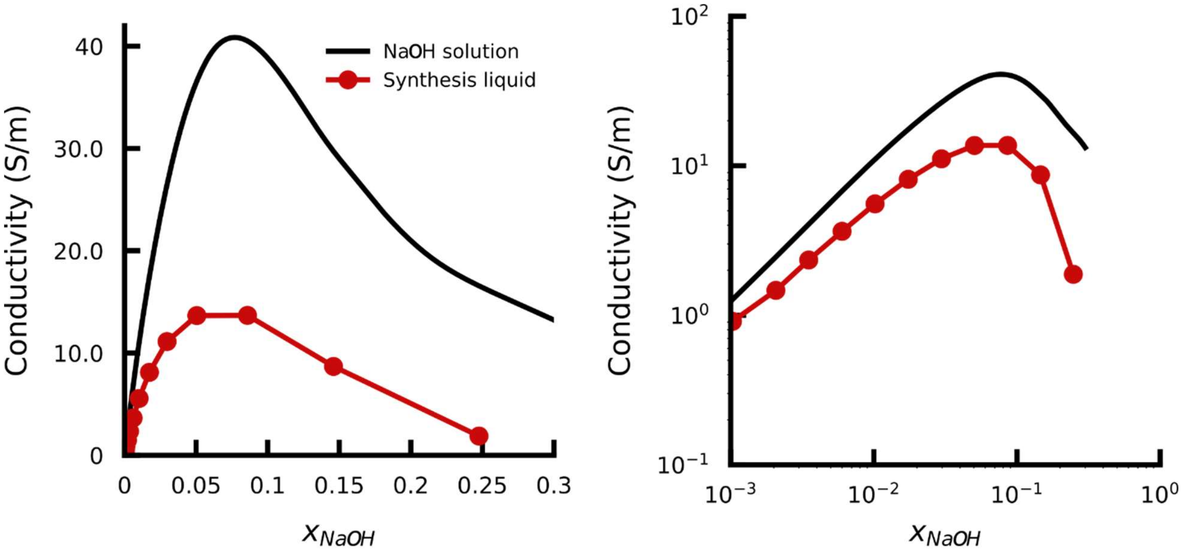


**Fig. S1 | Conductivity comparison between NaOH solutions and HSIL zeolite synthesis liquids of equal nominal charge density, $x_{NaOH}$**

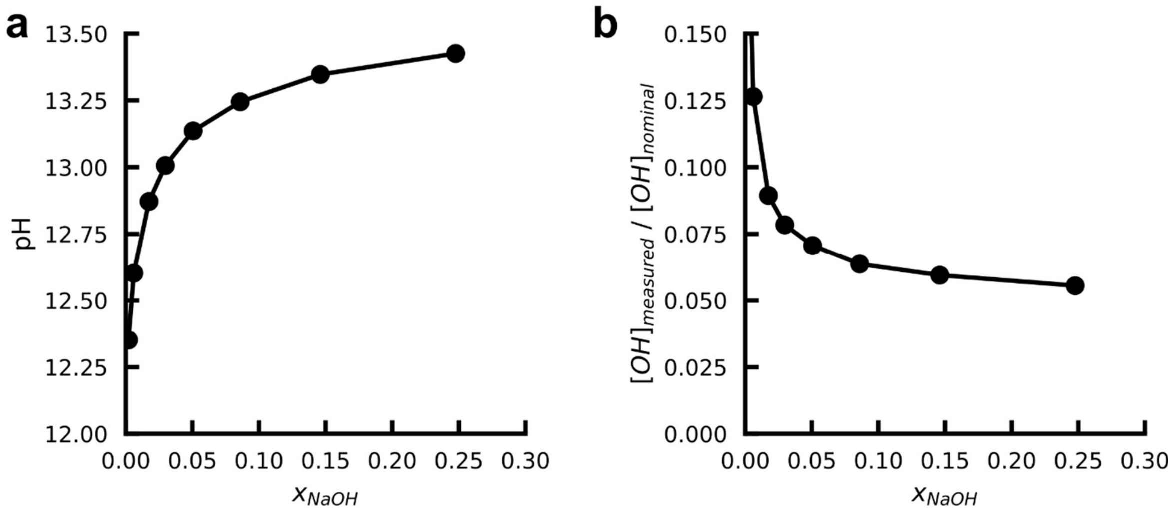


**Fig. S2 | Hydrogen electrode pH measurements of HSIL zeolite synthesis liquids. a,** pH data directly probed via the pH-dependent oxidation of molecular hydrogen for synthesis liquids of variable water content. **b,** Relative activity of hydroxide ions in comparison with their nominal concentration. Note that $x_{Si+Al}$ / $x_{NaOH}$ = 0.5, and therefore hydroxide ion depletion due to the presence of (alumino)silicate species remains constant despite a changing $x_{NaOH}$.

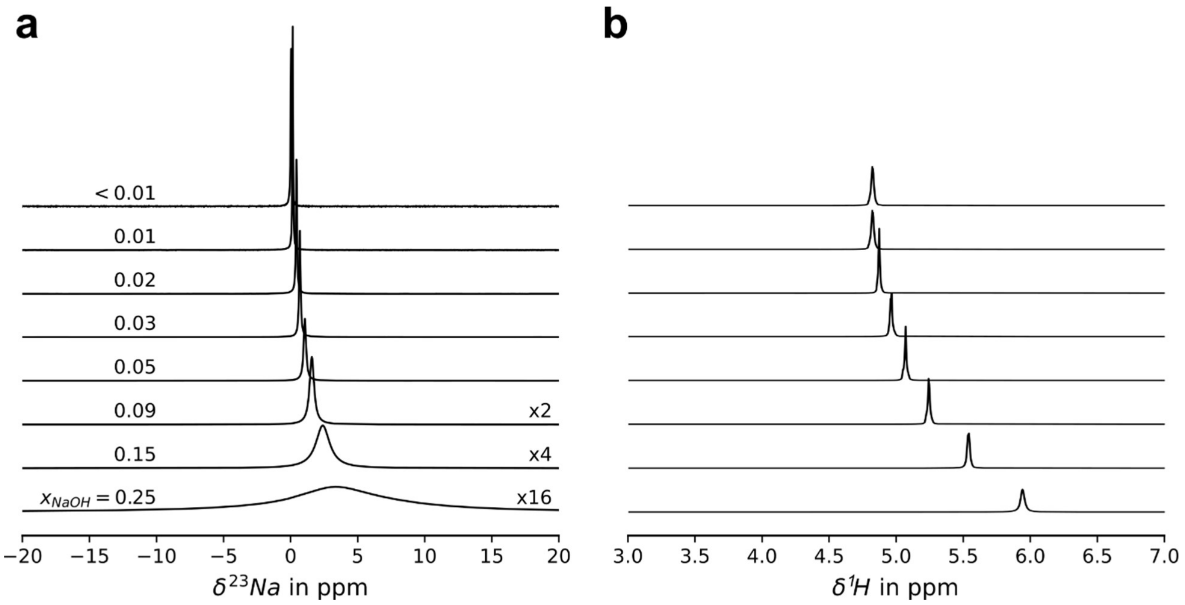


**Fig. S3 | Overview of the $^{1}$H and $^{23}$Na MAS NMR measurements. a,** $^{23}$Na measurements, intensity-scaled for plotting purposes, with the indicated factors. **b,** $^{1}$H measurements. All plots were normalized based on the total curve area.

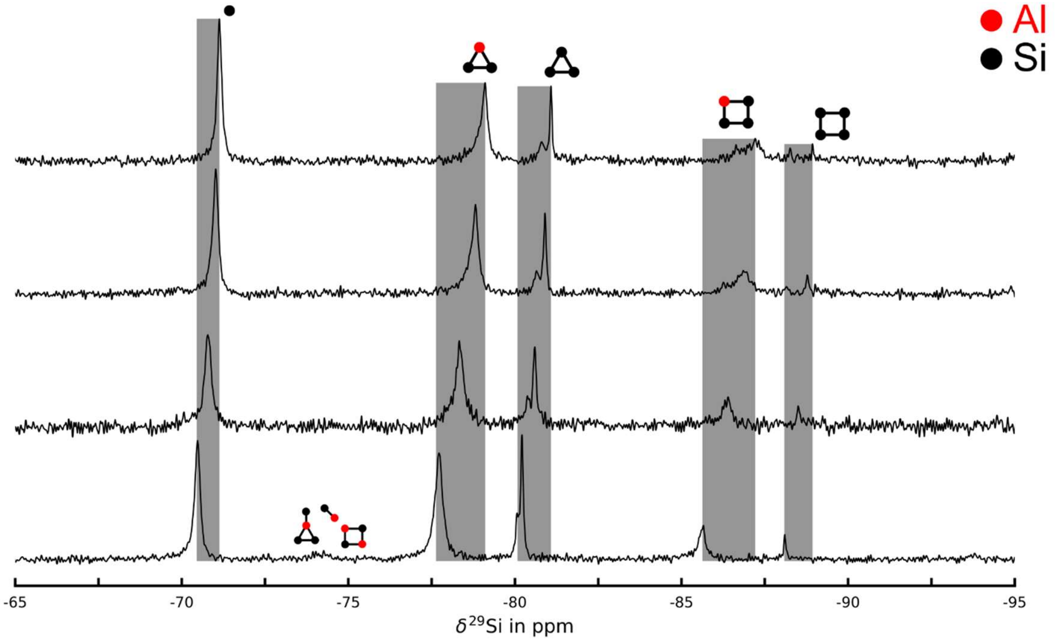


**Fig. S4 | $^{29}$Si MAS NMR measurements**. The measurements reveal that 6 categories of silicate contributions exist. Based on observations of silicate oligomerisation in sodium silicate solutions, monomeric, 3-ringed, and 4-ringed silicate oligomers are identified[9]. Prior literature on potassium aluminosilicate solutions[1,10] shows 2 additional oligomeric contributions belonging to aluminosilicate dimers, branched and unbranched 3-rings.

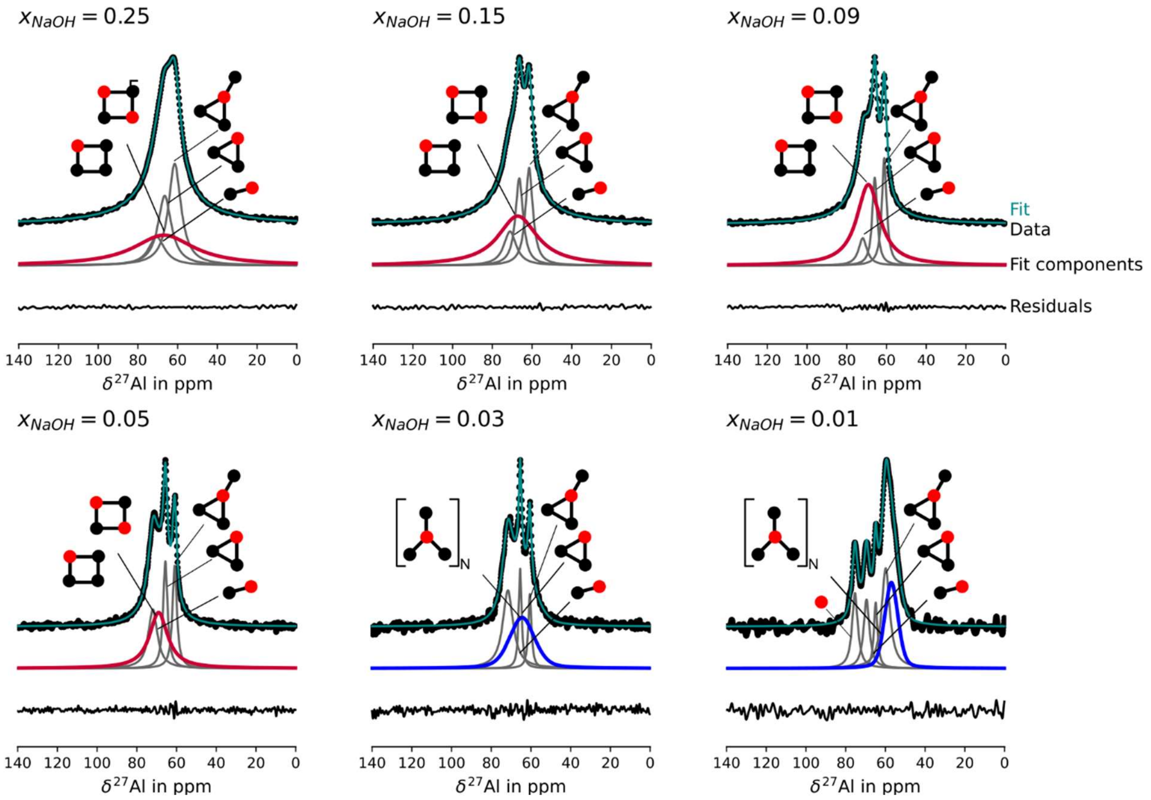


**Fig. S5| $^{27}$Al MAS NMR measurements**.

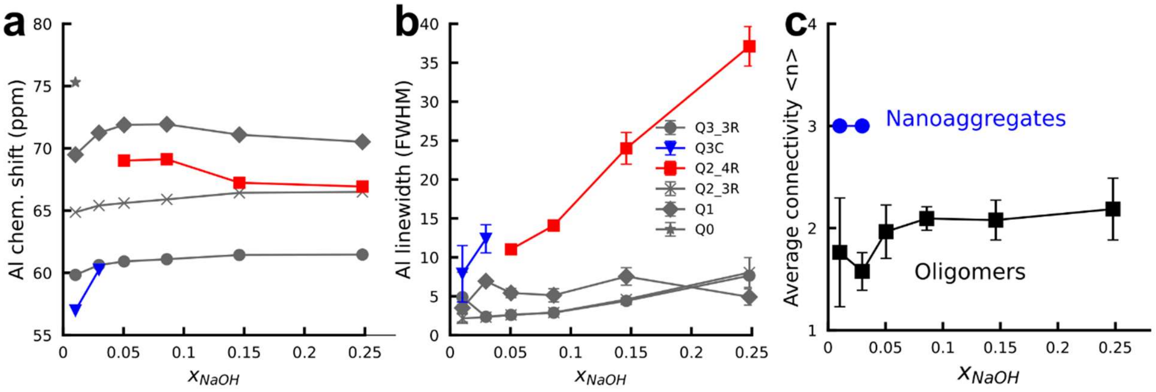


**Fig. S6 | Fitting parameter overview of $^{27}$Al NMR deconvolution. a,** Fitted chemical shifts. **b,** Fitted linewidths. **c,** Observed trend in average connectivity.

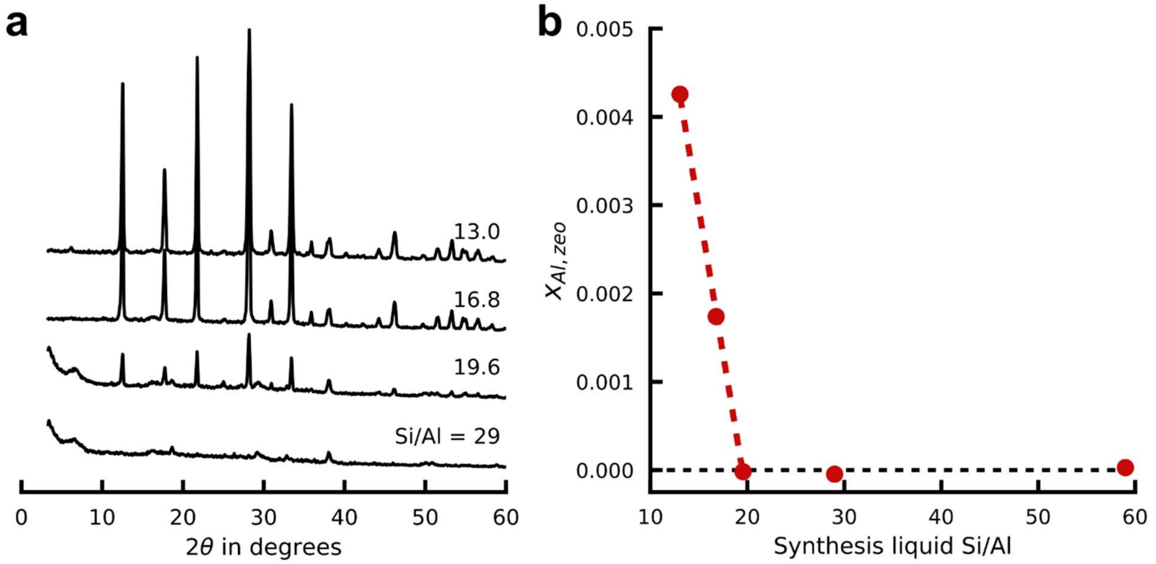


**Fig. S7 | Supersaturation of ion association complexes initiate GIS-type zeolite formation a,** XRD patterns of the recovered solids of sample composition $x_{NaOH}$=0.15 with variable aluminate content, given by 0.5 $Si(OH)_4$ : [0.5 / (Si/Al)] $Al(OH)_3$ : 1 NaOH : 5 $H_2O$ Note that the samples with highest Si/Al did not synthesize any solid. Therefore, the XRD pattern is not included. **b,** Quantitative yield analysis via ICP-OES reveals the supersaturation point of aluminosilicate ion-association complexes.

# 1. Supplemental Figures and Tables

**Table S1 | Overview of employed absorption correction factors muR.** X-ray diffraction measurements were performed in glass capillaries with an internal diameter of 0.35mm and muR was calculated based on a water-free elemental composition of $Si_aAl_1O_{2+2a}Na_1$, with the solid Si/Al ratio, measured via ICP-OES.

| Sample | Packing Density (g/cc) | Solid Si/Al ratio | Abs. Corr. muR |
|---|---|---|---|
| 0.25 | 0.316 | 1.29 | 0.34 |
| 0.15 | 0.594 | 1.50 | 0.65 |
| 0.09 | 0.661 | 1.65 | 0.72 |
| 0.05 | 0.758 | 1.82 | 0.84 |
| 0.03 | 0.213 | 1.93 | 0.24 |
| 0.02 | 0.341 | 2.15 | 0.38 |
| 0.01 | 0.293 | 2.17 | 0.32 |

**Table S2 | Overview of the quantitative zeolite synthesis yield analysis.** The fractional aluminate content of corresponding synthesis liquids ($x_{Al,}$) and mother liquors ($x_{Al,ML}$) yield the solid aluminate yield ($x_{Al,solid}$), as determined via ICP-OES measurements.

| Sample | $x_{Al}$ | $x_{Al,ML}/x_{Al}$ | $x_{Al,ML}$ | $x_{Al,solid}$ |
|---|---|---|---|---|
| 0.25 | 0.00619 | 0.667 | 0.00413 | 0.00206 |
| 0.15 | 0.00365 | 0.543 | 0.00198 | 0.00167 |
| 0.09 | 0.00215 | 0.458 | 0.00098 | 0.00117 |
| 0.05 | 0.00126 | 0.353 | 0.00044 | 0.00082 |
| 0.03 | 0.00074 | 0.289 | 0.00021 | 0.00053 |
| 0.02 | 0.00043 | 0.156 | 0.00006 | 0.00037 |
| 0.01 | 0.00015 | 0.052 | 0.00001 | 0.00014 |

**Table S3 | Overview of the hydrogen pH measurement results and analysis.**

| $x_{NaOH}$ | $E_{rel}\ (mV)$ | $pH$ | $[OH^{-}]_{meas}\ (mol/kg)$ | $[OH^{-}]_{nom}\ (mol/kg)$ |
|---|---|---|---|---|
| 0.25 | -376.9 | 13.42 | 0.411 | 7.40 |
| 0.15 | -372.4 | 13.35 | 0.320 | 5.38 |
| 0.09 | -366.4 | 13.25 | 0.234 | 3.67 |
| 0.05 | -360.0 | 13.14 | 0.169 | 2.39 |
| 0.03 | -352.4 | 13.00 | 0.117 | 1.50 |
| 0.02 | -344.5 | 12.87 | 0.082 | 0.91 |
| 0.01 | -328.7 | 12.60 | 0.041 | 0.33 |
| <0.01 | -314.0 | 12.35 | 0.023 | 0.12 |

**Table S4 | Overview of the ion pairing chemical equilibrium model results.** The fraction of sodium in ion pairs with (alumino)silicate anions, based on the chemical shifts and exchange modelling Are given by $f_{IP,\ \delta}$ and $f_{IP,\Delta v,}$, respectively.

| $x_{NaOH}$ | $\delta_{obs}\ (ppm)$ | $f_{IP\_\delta}$ | $f_{IP\_\Delta v}$ |
|---|---|---|---|
| 0.25 | 3.38 | 0.525 | 0.525 |
| 0.15 | 2.40 | 0.383 | 0.350 |
| 0.09 | 1.59 | 0.269 | 0.226 |
| 0.05 | 1.06 | 0.182 | 0.150 |
| 0.03 | 0.69 | 0.118 | 0.096 |
| 0.02 | 0.44 | 0.074 | 0.059 |
| 0.01 | 0.16 | 0.045 | 0.018 |
| <0.01 | 0.04 | 0.0163 | 0.0001 |

**Table S5 | Overview of the $^{27}$Al NMR fitting parameters for the NaOH containing dilution series.** Fitting errors are calculated at 95% confidence.

| Sample | Contrib. | Peak shape | $\delta(^{27}Al)$ | | FWHM | | Amp. | |
|---|---|---|---|---|---|---|---|---|
| | | | value | error (%) | value | error (%) | value | error (%) |
| 0.25 | Q1 | lor | 70.52 | 1.96 | 4.91 | 21.35 | 2.64 | 93.83 |
| | Q2_4R | lor | 66.93 | 0.60 | 37.10 | 6.82 | 10.62 | 6.93 |
| | Q2_3R | lor | 66.50 | 0.42 | 8.05 | 23.65 | 5.30 | 51.70 |
| | Q3_3R | lor | 61.47 | 0.23 | 7.63 | 4.74 | 7.32 | 13.82 |
| 0.15 | Q1 | lor | 71.09 | 0.39 | 7.54 | 14.88 | 2.43 | 34.09 |
| | Q2_4R | lor | 67.24 | 0.44 | 24.00 | 8.47 | 11.19 | 9.35 |
| | Q2_3R | lor | 66.43 | 0.08 | 4.59 | 4.97 | 3.77 | 8.12 |
| | Q3_3R | lor | 61.44 | 0.06 | 4.41 | 3.82 | 4.06 | 6.47 |
| 0.09 | Q1 | lor | 71.93 | 0.25 | 5.13 | 16.57 | 1.33 | 35.25 |
| | Q2_4R | lor | 69.12 | 0.31 | 14.09 | 3.15 | 10.75 | 5.52 |
| | Q2_3R | lor | 65.90 | 0.04 | 2.91 | 4.13 | 2.41 | 0.04 |
| | Q3_3R | lor | 61.10 | 0.02 | 2.89 | 2.22 | 2.93 | 2.53 |
| 0.05 | Q1 | lor | 71.88 | 0.17 | 5.42 | 10.96 | 3.02 | 28.13 |
| | Q2_4R | lor | 69.02 | 0.98 | 11.03 | 4.45 | 5.81 | 13.29 |
| | Q2_3R | lor | 65.61 | 0.04 | 2.61 | 3.84 | 2.63 | 6.07 |
| | Q3_3R | lor | 60.92 | 0.02 | 2.61 | 3.01 | 2.64 | 6.07 |
| 0.03 | Q1 | lor | 71.24 | 0.10 | 6.94 | 2.78 | 6.52 | 3.15 |
| | Q2_3R | lor | 65.41 | 0.04 | 2.32 | 3.94 | 2.57 | 3.88 |
| | Q3_3R | lor | 60.63 | 0.05 | 2.37 | 8.37 | 1.84 | 11.67 |
| | Q3_C | 62% lor<br>38% gau | 60.26 | 0.73 | 12.38 | 14.55 | 2.279 | 16.49 |
| 0.01 | Q0 | lor | 75.30 | 0.21 | 3.42 | 15.53 | 2.43 | 13.52 |
| | Q1 | lor | 69.49 | 0.29 | 3.50 | 22.71 | 2.27 | 24.86 |
| | Q2_3R | lor | 64.87 | 0.25 | 2.16 | 28.74 | 1.34 | 30.24 |
| | Q3_3R | lor | 59.84 | 0.65 | 4.89 | 64.99 | 4.62 | 98.33 |
| | Q3_C | 38% lor<br>62% gau | 56.99 | 5.49 | 7.89 | 46.02 | 4.95 | 83.30 |

**Table S6 | Mass recipes of the studied samples**

| $x_{NaOH}$ | $m_{H_2O}\ (g)$ | $m_{NaOH}\ (g)$ | $m_{Al(OH)_3.H_2O}\ (g)$ | $m_{Na-HSIL}\ (g)$ |
|---|---|---|---|---|
| 0.25 | 5.697 | 4.555 | 0.564 | 19.169 |
| 0.15 | 12.340 | 3.310 | 0.410 | 13.930 |
| 0.09 | 17.954 | 2.258 | 0.280 | 9.502 |
| 0.05 | 22.183 | 1.465 | 0.181 | 6.166 |
| 0.03 | 25.107 | 0.917 | 0.114 | 3.860 |
| 0.02 | 27.011 | 0.560 | 0.069 | 2.358 |
| 0.01 | 28.931 | 0.200 | 0.025 | 0.843 |
| <0.01 | 29.627 | 0.070 | 0.009 | 0.294 |
